# Beneficial Effects of Walnut Oligopeptides on Muscle Loss in Senescence-Accelerated Mouse Prone-8 (SAMP8) Mice: Focusing on Mitochondrial Function

**DOI:** 10.3390/nu14102051

**Published:** 2022-05-13

**Authors:** Rui Fan, Yuntao Hao, Qian Du, Jiawei Kang, Meihong Xu, Yong Li

**Affiliations:** 1 Department of Nutrition and Food Hygiene, School of Public Health, Peking University, Beijing 100191, China; fanruirf@bjmu.edu.cn (R.F.); haoyuntaolly@163.com (Y.H.); duqian_1991@163.com (Q.D.); kangjwdt@163.com (J.K.); 2 Beijing Key Laboratory of Toxicological Research and Risk Assessment for Food Safety, Peking University, Beijing 100191, China

**Keywords:** walnut oligopeptides, SAMP8, mitochondrial function, muscle loss, anti-inflammatory

## Abstract

Aging-related muscle loss is a hallmark of aging and is the cause of some negative outcomes. An optimized diet and supplements have a positive effect in slowing down the process of muscle loss. This study was designed to evaluate the beneficial effects of walnut oligopeptides (WOPs) on aging-related muscle loss and explore the possible underlying mechanism in Senescence-Accelerated Mouse Prone 8 (SAMP8) Mice. SAMP8 mice were randomly divided into four groups (*n* = 15/group), including one group which was the SAMP8 age control group and three groups those were WOP intervention groups. Meanwhile, Senescence Accelerated Resistant Mouse 1 (SAMR1) mice (*n* = 12), which had normal senescence rates, were used as model controls. During the six-month intervention period, the age control and normal control groups were given sterilized water, while the three WOP intervention groups were given WOP solution with low (110 mg/kg·bw), medium (220 mg/kg·bw) and high concentrations (440 mg/kg·bw), respectively. The results showed that WOPs could significantly increase muscle mass and improve physical performance (wire hang and catwalk behavioral tests) in aging mice. Moreover, WOPs could significantly reduce the levels of IL-1β, IL-6 and TNF-α in serum and gastrocnemius tissues and increase the mitochondrial DNA content, as well as the expression levels of AMPK, PGC-1α, NRF-1 and TFAM in the gastrocnemius muscle of aging mice, which was speculated to be the specific mechanism related to mitochondrial function improvement and inflammation reduction. These results indicate that WOPs can improve aging-related muscle loss, in term of both muscle mass and physical performance, and WOP supplements seems to be potentially effective in elderly individuals.

## 1. Introduction

As life expectancy increases, the world is experiencing a dramatic aging process [1]. According to WHO data, the number of elderly people (aged 60 and above) is expected to exceed 1.4 billion in 2030 and 2.1 billion in 2050 [2]. For these individuals, aging leads to degenerative changes that occur at multiple levels, from the molecular metabolism to organ systems, which contribute to the manifestation of various health problems [3]. As the largest motor organs in the body, the loss of the mass, size, strength and function of skeletal muscles, is an age-related hypofunction. The age-related hypofunction of skeletal muscles compromises the physical and social functional abilities of elderly individuals. Moreover, a progressive loss of muscle and function could lead to sarcopenia (diagnosis code ICD-10-CM), which reduces mobility levels and raises the likelihood of adverse outcomes such as falls, fractures and death [4,5,6]. Intending to enable earlier interventions, the Asian Working Group for Sarcopenia (AWGS) 2019 introduced the concept of “possible sarcopenia” [7]. According to this diagnostic criterion, the prevalence of possible sarcopenia, sarcopenia and severe sarcopenia were 38.5%, 18.6% and 8.0%, respectively, among the elderly Chinese population [8]. The prevalence of sarcopenia was 25.7% in Asia [7], while according to the EWGSOP (European Working Group on Sarcopenia in Older People) definition, the prevalence of sarcopenia was 1–29% in community dwelling populations where residents were aged ≥ 50 years [9], and 14–33% of residents required long-term care [10]. In 2000, the direct economic costs associated with sarcopenia were reported to be approximately USD 18.5 billion in the USA [10]. A report concerning the economic impact of financial stress in Chinese households highlighted that severe sarcopenia may increase the risk of CHE (catastrophic health expenditure) (odds ratio: 1.04, 95% CI: 1.01–1.07, *p* < 0.01) [11]. Therefore, this disease presents huge personal and social burdens. A consensus has been reached that timely and effective interventions for moderate and severe sarcopenia can contribute to reduce the health burden the disorder presents, and the AWGS 2019 report recommends individualized lifestyle interventions to enable greater awareness of sarcopenia prevention and improvement strategies.

Nutritional supplementation and resistance training are still the primary strategies used in both the prevention and treatment of sarcopenia across the life course [10]. A number of studies demonstrated that if older adults with sarcopenia consumed sufficient dietary protein, the muscles mass and physical performance would be improved, either with ordinary food intake or with supplementation of products, such as whey protein and leucine [12,13,14]. From clinical trials [15,16,17,18], animal experiments [19] and cell cultures [20,21], accumulating evidence shows that certain protein intervention can delay or reduce symptoms of sarcopenia and improve biomarkers in the disorder. 

Currently, bioactive peptides are widely used as optimal food ingredients due to their high absorptivity, hypo allergenicity, diverse biological activity, etc. [22]. Considered to be protein-upgrading products, hydrolysates or peptides play a beneficial role in sarcopenia [23]. As a typical bioactive peptide, walnut (*Juglans regia* L.) oligopeptides (WOPs) are enzymatically hydrolyzed from walnut seed residues after oil extraction. Previously, our studies have suggested that WOPs exhibit practical beneficial effects beyond their nutritional profiles, such as anti-fatigue, anti-hypoxia, regulating blood lipids, promoting probiotics proliferation, and reducing systematic inflammatory, oxidative stress and metabolic disorders, as well as other biological activity including the maintenance of mitochondrial function [24,25,26,27,28]. Moreover, our results show that the amino acid composition of WOP samples is rich in arginine (Arg) > phenylalanine (Phe) > leucine (Leu) > alanine (Ala) > glutamic acid (Glu) [27,28], of which, essential amino acids, especially branch-chain amino acids (BCAA), are good dietary choices for the elderly individuals, as they enable muscle protein synthesis [29,30]. Therefore, it can be speculated that WOPs might have the potential benefits for skeletal muscle. 

SAMP8 (Senescence Accelerated Prone Mouse 8) mice, developed from the AKR/J strain, are a rapid aging mouse model, which have the advantage of having short lifespans and experiencing accelerated aging processes. To compare SAMP8 mice with an accelerating senescence rate, SAMR1 (Senescence Accelerated Resistant Mouse 1) are usually utilized [31,32,33]. Compared with SAMR1mice, it has been reported that SAMP8 mice showed decreased muscle strength, muscle fiber size and muscle phosphocreatine level with aging [34]. Studies have shown that the muscle mass of SAMP8 mice reaches its peak at the age of seven months, and muscle loss occurs in the eighth month of life for SAMP8 mice, which causes a significant decrease in muscle mass (12.41%), muscle strength (11.64%) and contractibility (25.96%) [35]. Additionally, mitochondrial dysfunction is the main cause of the high oxidative stress state in SAMP8 mice and is related to aging and sarcopenia [36,37]. Therefore, SAMP8 can be recommended as a cost-effective animal model for sarcopenia research. Thus, the present study was performed to investigate the impacts of WOPs on age-related muscle loss and explore the possible underlying mechanism via a mitochondrial target in SAMP8 mice. 

## 2. Materials and Methods

### 2.1. Preparation of WOPs Sample

The character of walnut oligopeptides (WOPs), provided by Beijing Huataitai biotechnology co. LTD, is faint yellow solid powder, and the components are main macromolecule peptides of which molecules are below 1000D. The amino acid composition of WOPs has already been shown in our previous study [26,27,28].

### 2.2. Animals and Treatments

In our study, 4-month-old male SAMP8 and SAMR1 mice, provided by the medical laboratory animal science department of Peking University, underwent adaptive feeding for 1 week before grouping. Fifteen SPF male SAMR1 mice were used as the SAMR1 model control group. Sixty SAMP8 mice were randomly divided into four groups according to body weights (15 mice per group), including the SAMP8-age control group, and three WOP intervention groups based on different WOP intervention doses. The mice were housed at a constant temperature (25 ± 1 °C) and humidity (50–60%) under a 12 h: 12 h light dark cycle. 

During the whole experimental period, standard food (American Institute of Nutrition Rodent Diets-93G (AIN-93G diet) was freely available to all of the mice. The SAMP8 age control group and the SAMR1 model control group were given ordinary drinking water, while the WOP intervention groups including SAMP8-WOPs-LG, SAMP8-WOPs-MG and SAMP8-WOPs-HG) were given low, medium and high concentrations of WOP solution (110 mg/kg·bw, 220 mg/kg·bw and 440 mg/kg·bw, respectively). We recorded their daily water intake and food intake, and measured their weights weekly. After 6 months of continuous intervention, the wire hang and catwalk behavioral tests were carried out. To reduce the level of systematic error and ensure the consistency of observation before and after the experiment, all of the tests were carried out by fixed personnel. During the whole process of the investigation, the movement of personnel was reduced, and silence was maintained in the test room. There were 2 days between the two behavioral tests to eliminate interferences.

For tissue collection, all of the mice were killed via cervical dislocation following eyeball blood sampling at the end of the experiment. The gastrocnemius tissue was separated and kept on ice immediately. 

### 2.3. Muscle Mass Measurement

To estimate the ratio of muscle mass at the total body level, an analysis using EchoMRI was performed according to the manufacturer’s instructions. After the animals were killed, the muscle was separated once immediately. The gastrocnemius, tibialis anterior, extensor digitorum longus, and soleus muscles were excised from both hindlimbs. The muscles from the right side were weighed, and frozen in liquid nitrogen. All tissue samples were stored at −80 °C until analysis. A part of these tissues was used for real-time PCR analysis. The muscle to body weight and gastrocnemius weight to body weight were calculated.

### 2.4. Wire Hang and Catwalk Test

The age-dependent physical performance of mice was tested, including wire hang and gait analysis (catwalk test). We tested the muscle strength of mice with suspension force in the wire hang test. In the wire hang test, the mouse was placed onto the top of a wire mesh cage, which was then gently inverted to encourage the mouse to grip the wire. The retention time was recorded. We set the maximum duration at 120 s. The average of the two times was recorded as the individual mouse muscle strength. 

CatWalk XT™ system (Noldus Information Technology, Wageningen, Netherlands) was used to assess the voluntary gait and locomotion of SAMP8 mice. The test was performed as according to the previous study [38]. For a gait test, the possible noncompliance with and refusal to complete running at gait was exclusive. Strict training was started one week prior to formal performance. The training and experimental procedures were performed during the same period of the day (from 8 a.m. to 2 p.m.) for one week. On training days, each animal was allowed to move at will along the corridor and freely explore the walkway. If the mice could not walk to the end or kept standing, a few food pellets were placed in the goal box as a motivator cue for a successful run. No noise was used to motivate the animal to run.

During the test, three compliant runs for each animal were acquired. Compliant runs were defined as run time ranging from 0.50 s to 7.0 s. Semi-automated labeling and analysis of paw prints with CatWalk XT^TM^ software provided static and dynamic gait parameters. 

### 2.5. Enzyme-Linked Immunosorbent Assay in Serum and Gastrocnemius

All the indicators were determined by assay kits, according to the protocol provided by the manufacturer, inflammatory parameters (IL-1β, IL-6, TNF-α) in serum and gastrocnemius.

### 2.6. Quantitative Real-Time PCR and Analyses of mtDNA Content in Gastrocnemius

Total RNA and DNA were isolated from gastrocnemius tissue of SAMP8 mice, using Trizol reagent (Invitrogen, Carlsbad, CA, USA) and DNeasy Tissue Kit (QIAGEN Sciences, Germantown, MD, USA), respectively. Real-time reverse transcription-PCR was performed to detect the RNA expression of target genes, as well as target mRNA values, and the content of mtDNA copy number was determined, according to our previous study [28]. The specific primers were listed as follows: AMPK Forward 5′-GAAAGTGAAGGTGGGCAAGC-3′ and Reverse 5′-GATGTGAGGGTGCCTGAACA-3′; SIRT1 Forward 5′-AGCGTCTTGACGGTAATCAA-3′ and Reverse 5′-AACTTGGACTCTGGCATGTG-3′; PGC-1α Forward 5′-TCACGTTCAAGGTCACCCTA-3′ and Reverse 5′-TCTCTCTCTGTTTGGCCCTT-3′; NRF-1 Forward 5′-CCATCTATCCGAAAGAGACAGC-3′ and Reverse 5′-GGGTGAGATGCAGAGTACAATC-3′; TFAM Forward 5′-CCTGAGGAAAAGCAGGCATA-3′ and Reverse 5′-TCACTTCGTCCAACTTCAGC-3′;mtDNA Forward 5′-CGTTAGGTCAAGGTGTAGCC-3′ and Reverse 5′-CCAGA CACACTTTCCAGTATG-3′; GAPDH Forward 5′-TGCCCCCATGTTTGTGATG-3′ and Reverse 5′-TGTGGTCATGAGCCCTTCC-3′. Cycling conditions were 95 °C for 5 min, followed by 40 repeats of 95 °C for 10 s and 60 °C for 30 s.

### 2.7. Statistical Analysis

Statistical analyses were performed using the SPSS software version 26 (SPSS Inc., Chicago, IL, USA). Data were expressed as mean ± standard deviation (SD) and analyzed by one-way analysis of variance (ANOVA) test; to determine the difference of parametric samples among groups, multiple comparisons of least significant difference (equal variances assumed) or the Dunnett’s T3 test (equal variances not assumed) were used. Survival curves of mice were estimated with the Kaplan–Meier analysis, and differences between groups were compared with the two-sided log-rank test; *p* < 0.05 indicated a statistically significant difference.

## 3. Results

### 3.1. Effects of WOPs on Body Composition and Gastrocnemius (%Body Weight) in Mice 

There was no significance in any of the changes to body weight, food and water consumption among the five groups.

As shown in Table 1, compared with the SAMR1-model control group, the lean mass (%body weight) and the gastrocnemius mass (%body weight) in the SAMP8 age control group significantly decreased (*p* < 0.05), suggesting that muscle attenuation progresses with aging. Compared with the SAMP8 age control group, the lean mass (%body weight) and gastrocnemius mass (%body weight) of mice in the WOP intervention group significantly increased (*p* < 0.05). There was a certain dose effect between different concentrations of WOP, but it was not significant (*p* > 0.05).

### 3.2. Effects of WOPs on the Physical Performance

**Figure 1:**
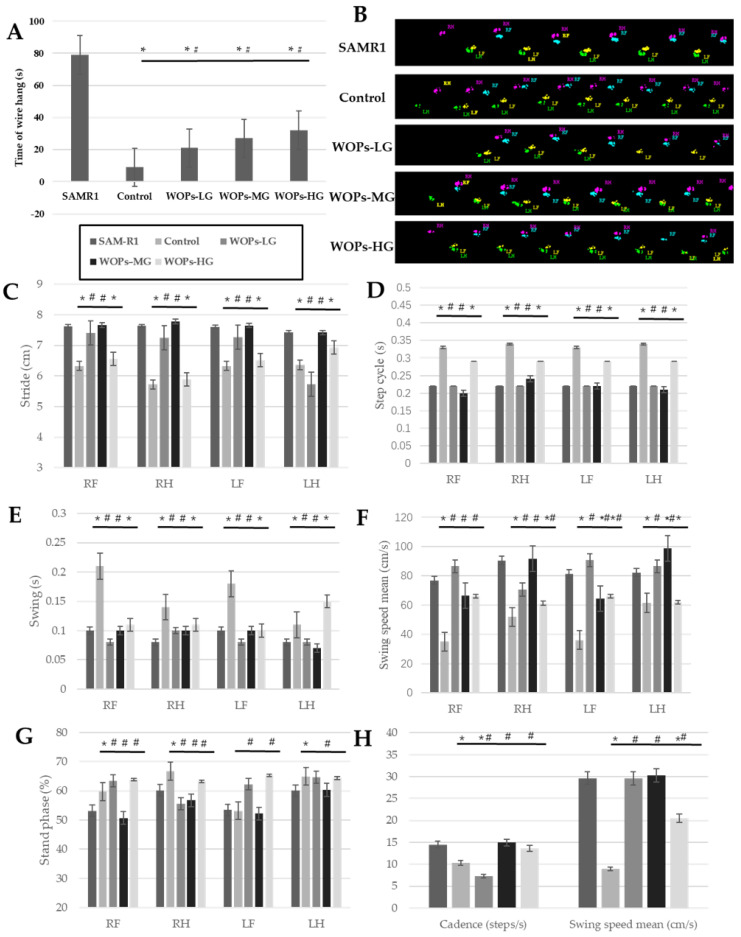
Effects of WOPs on the physical performance. (**A**) Wire hang test; (**B**–**H**) catwalk test. Data are expressed as mean ± SD (*n* = 8 per group). * *p* < 0.05 vs. SAMR1 and ^#^ *p* < 0.05 vs. Control. SAMR1, SAMR1 model control group; Control, SAMP8 age control group; WOPs-LG, SAMP8-WOPs-LG at a dose of 110 mg/kg·bw; WOPs-MG, SAMP8-WOPs-MG at a dose of 220 mg/kg·bw; WOPs-HG, SAMP8-WOPs-HG at a dose of 440 mg/kg·bw.

As shown in Figure 1A, the mice in the SAMR1 model control group spent the longest time on the wire hang test, 8.16 times longer than the mice in the SAMP8 age control group. Meanwhile, the time taken by of all of the mice in the WOP intervention groups was also significantly lower than that of the SAMR1 model control group (*p* < 0.05), suggesting that muscle strength decreased significantly with aging, indicating the decrease in muscle function and muscle attenuation. Compared with the SAMP8 age control group, the time taken by mice in the WOP intervention groups significantly increased (*p* < 0.05), and there was a certain dose-effect among different concentrations of WOPs, but no significant differences were observed, suggesting that the WOP intervention had a positive effect on age-related muscle function reduction. 

The results of the gait change trend are shown in Figure 1B–H, Among the four limbs (Figure 1C), the stride lengths of the mice in the SAMP8 age control group exhibited an obviously smaller level than that in the SAMR1 normal control group (*p* < 0.05), while the relatively bigger one in the stride lengths were observed in the WOPs intervention groups, of which the right limbs and left forelimb of mice in the SAMP8-WOPs-LG and SAMP8-WOPs-MG were significantly higher than that of the SAMP8 age control group (*p* < 0.05). 

For the value of step cycle (Figure 1D), the mice in the SAMP8 age control group showed the biggest values among the four limbs (*p* < 0.05), while the WOP intervention with the low and median dose could significantly decrease the step value of aging mice (*p* < 0.05). On the contrary, the relatively lower values of the cadence in the SAMP8 age control group and SAMP8-WOPs-LG MOP was seen from Figure 1H, while the mice treated by median and high dose WOP exhibited a significant increase (*p* < 0.05). 

From the Figure 1E,F, the change trends of swing time and swing speed were opposite. The swinging times of four limbs in the SAMR8 age control group were significantly longer than those in other groups, including the SAMR1 normal control group, SAMP8-WOPs-LG and SAMP8-WOPs-MG (*p* < 0.05); at the same time, the right limbs and left forelimb of mice in the SAMR8 age control group showed a significantly smaller swing speed than those in the other four groups (*p* < 0.05). Overall, the swing speed mean exhibited the same change trend (Figure 1H). For stand phase property, the hind limbs of mice in three WOP intervention groups showed a relatively lower value than those in the SAMP8 age control group, of which the decrease in the right hind limb exhibited a significant difference (*p* < 0.05); while, for the forelimbs’ stand phase, the mice in the SAMP8-WOPs-HG exhibited a relatively lower result than those in the SAMP8 age control group, of which the right forelimb showed a significant decrease (*p* < 0.05). As seen from the Figure 1B, the gait patterns observed in the SAMP8 age normal group and the SAMR1 normal control group were rotary pattern and cruciate pattern, respectively, while the alternative pattern was seen in the three WOP intervention groups.

The above results suggest that WOPs can improve muscle mass and muscle function of SAMP8 mice, thus improving the physical performance of SAMP8 mice.

### 3.3. Effects of WOPs on Proinflammatory Factors in Serum and Gastrocnemius

**Figure 2:**
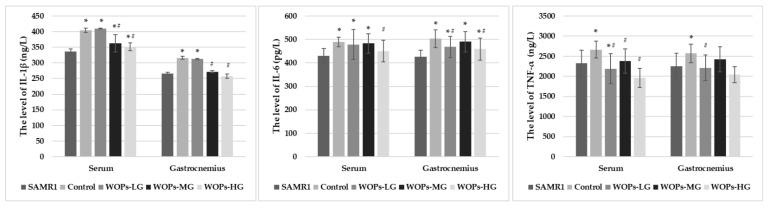
Effects of WOPs on the proinflammatory factors in serum and gastrocnemius. Data is expressed as mean ± SD (*n* = 13–15 per group). *p* values were assessed using ANOVA-test. * *p* < 0.05 vs. SAMR1 and ^#^ *p* < 0.05 vs. Control. SAMR1, SAMR1 model control group; Control, SAMP8 age control group; WOPs-LG, SAMP8-WOPs-LG at a dose of 110 mg/kg·bw; WOPs-MG, SAMP8-WOPs-MG at a dose of 220 mg/kg·bw; WOPs-HG, SAMP8-WOPs-HG at a dose of 440 mg/kg·bw.

As the results show in Figure 2, IL-1β and IL-6 levels in serum increased significantly in the SAMP8 age control group and WOP intervention groups in comparison with the SAMR1 normal control group (*p* < 0.05), suggesting a chronic inflammatory state occurs in vivo with aging. Compared with the SAMP8 age control group, in WOP intervention groups, the IL-1β, IL-6 and TNF-α levels in serum were decreased, especially SAMP8-WOPs-MG and SAMP8-WOPs-HG (*p* < 0.05). There was a certain dose effect between different concentrations of WOPs, but there was no significant difference. 

IL-1β, IL-6 and TNF-α levels in gastrocnemius muscle tissue were also examined. Compared with SAMR1 mice, SAMP8 mice in the age control group and the WOP intervention groups had relatively higher levels of IL-1β, IL-6 and TNF-α in gastrocnemius, of which the IL-6 levels of SAMP8 mice showed the significant increase (*p* < 0.05), suggesting muscle loss occurred with aging, related to the chronic inflammatory infiltrate state. Compared with the SAMP8 age control group, IL-1β, IL-6 and TNF-α levels in the gastrocnemius tissue of the all of the WOPs intervention groups relatively decreased (*p* < 0.05), with an especially significant decrease in IL-1β and IL-6 levels shown in the SANP8 WOPs-HG (*p* < 0.05), as well as in TNF-α level as shown in the SAMP8 WOPs-LG. It seems that there was a certain dose effect among different concentrations of WOPs, but no significant difference was observed.

### 3.4. Effects of WOPs on Mitochondrial Function Factors and mtDNA Content in the Gastrocnemius

**Figure 3:**
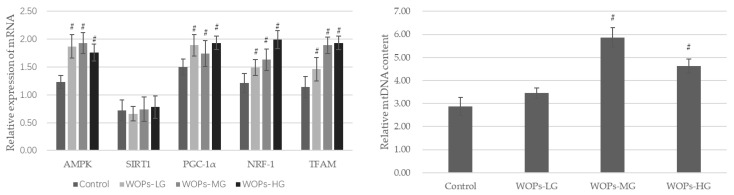
Effects of WOPs on the RNA expression of AMPK, SIRT1, PGC-1α, NRF-1, TFAM, and relative mtDNA content in gastrocnemius by real-time PCR analysis. Data are expressed as mean ± SD (*n* = 6 per group). ^#^ *p* < 0.05 vs. Control. Control, SAMP8 age control group; WOPs-LG, SAMP8-WOPs-LG at a dose of 110 mg/kg·bw; WOPs-MG, SAMP8-WOPs-MG at a dose of 220 mg/kg·bw; WOPs-HG, SAMP8-WOPs-HG at a dose of 440 mg/kg·bw.

As shown in Figure 3, the mRNA expressions of AMPK, PGC-1α, NRF-1 and TFAM, which are mitochondrial biogenesis factors considered essential for mitochondrial gene expression in mammals, were markedly increased in the WOP intervention groups compared with the SAMP8 age control group (*p* < 0.05). Additionally, the mtDNA content was also significantly improved after WOP treatment with the median and high dose (*p* < 0.05). 

## 4. Discussion

Sarcopenia is a hallmark of aging and is the cause of many negative outcomes. In the present study, SAMP8 mice were used as an aging model. Walnuts, consumed worldwide, are a kind of nut with a variety of health benefits. In the past, the benefits of walnuts were mostly attributed to walnut oil, and little attention was paid to WOPs. The present study shows that WOPs can significantly improve aging-related muscle loss and improve physical performance in aging mice. Specific mechanisms include improving mitochondria function via AMPK/SIRT1/PGC-1α/NRF-1/TFAM, reducing the expression of inflammatory factors. All of the findings mentioned above indicate that WOPs have the potential to improve sarcopenia. It suggested that WOPs would be optimal supplementations in sarcopenia. 

The function and performance of the neuromuscular system decline significantly as the human body progresses from maturity to aging. During the aging process, muscle wasting occurs, which is typically manifested as a decline in muscle mass, strength and physical performance. The decline in physical strength is due to insufficient protein synthesis in skeletal muscle, impaired oxidative phosphorylation after cell structure destruction, and reduced ATP synthesis. In this study, body composition and gastrocnemius wet weights were calculated to evaluate muscle mass. Meanwhile, the muscle weight/body weight ratio was calculated. Compared with the age control, the data showed that lean mass (%body weight) and muscle wet weight were significantly increased in the WOP intervention groups. In addition, this indicated that WOP supplementation has the potential to slow down the loss of muscle mass and could significantly provoke an increase in the weight of aging gastrocnemius, as well as gastrocnemius index (%body weight). 

To assess the functional consequences of WOPs for skeletal muscle loss, the hanging wire and catwalk test were carried out. The catwalk gait detection method is an automated and computerized gait analysis technology which can be used to objectize and digitize a large number of dynamic gait parameters and comprehensively and quantitatively analyze the gait function of mice in a short time. Additionally, it can be used to analyze various gait indicators more objectively and accurately with the help of computer analysis. The catwalk method has previously been applied to the functional evaluation of spinal cord injury models [39]. In the present study, the catwalk method was used to evaluate the gait of mice in all of the groups. 

Compared with SAMP8 without WOP treatment, it was clearly observed that WOP intervention could improve the swing speed, stride length, cadence and gait patterns, and also decrease step cycle and swing time, suggesting the beneficial effect of WOPs on power and coordination. Meanwhile, the wire hang test was carried out. It was observed that the WOP intervention groups displayed significant increases in grip compared with the SAMP8 age control group. Although there were no markable differences in the time of latency to fall among the WOP intervention groups, it could be seen that the mice in the WOP intervention groups fell later than those in the SAMP8 age control group. The mass and physical functional results suggest that WOPs have the potential to improve aging-related muscle disorders.

A chronic inflammatory state is one of the characteristics of aging and one of the risk factors for the high incidence of various age-dependent chronic diseases [8,31,40]. High levels of proinflammatory cytokines were associated with the loss of muscle mass and poor physical function in older adults. Therefore, age-related chronic low-grade inflammation could be a vital contributor to sarcopenia [41,42]. We demonstrated that WOPs could reduce the level of TNF-α, IL-1 β and IL-6 in the serum of aging mice [43,44]. These findings are highly relevant for elderly and frail individuals or people who often experience inflammation and loss of muscle quality. Additionally, studies have confirmed that an increase in the intake of components with antioxidant and anti-inflammatory properties from diet or from supplementation could effectively prevent the negative impact of antioxidant deficiency, including oxidative status, lower muscle strength and muscle fatigue after an exercise challenge [14,21,45,46,47]. As typical bioactive peptides, WOPs have been shown to have great anti-inflammatory and antioxidative biological activities [24,25,26,27,28]. Combined with the results of behavioral experiments, it can be speculated that WOPs can reduce the overexpression of age-related inflammatory factors and reduce the chronic inflammatory state of muscle tissue in aging individuals, thereby reducing the occurrence of related inflammation-dependent muscle dysfunction.

Mitochondria are one of the most important organelles; they are distributed in myocytes and produce ATP to support the normal physiological activity of myocytes. Numerous investigations have revealed age-related mitochondrial alterations [38,48,49]. Mitochondria also play an important regulatory role in oxidative stress [50]. Consequently, mitochondrial dysfunction is linked to muscle loss and even sarcopenia. In this study, AMPK, SIRT1, PGC-1, NRF-1, TFAM and mtDNA copy numbers were measured to evaluate mitochondrial function. PGC-1α is a transcriptional co-activator involved in mitochondrial biogenesis and is closely related to the occurrence and development of sarcopenia [49]. The upregulation of the gene expression of PGC-1α can regulate the biogenesis and electron transport systems of mitochondria [51]. AMPK is an upstream target of a series of phosphorylation-dependent adaptive modifiers, including PGC-1α, which can activate catabolic pathways to generate ATP while deactivating energy-consuming anabolic processes. NRF-1 is a positive regulator of transcription, which initiates the synthesis of mitochondrial proteins, such as TFAM. Additionally, TFAM is a direct regulator of mtDNA duplication [52]. mtDNA copy number was considered as a surrogate marker of mitochondrial function [53]. NRF-1, TFAM and mtDNA represent the dysregulation of mitochondrial quality control processes and contribute to sarcopenia [54]. The results discussed above implied a protective role of WOPs through promoting mitochondrial biogenesis via the AMPK/PGC-1/NFR-1/TFAM signaling pathway. Research has found that increasing ATP levels in C2C12 myotubes enhanced the expression of mitochondrial biogenesis, including NRF-1, TFAM and PGC-1α [55]. In the present study, we found that WOPs could improve mitochondrial function in gastrocnemius tissue by restoring mtDNA content and increasing the mRNA expression of AMPK, PGC-1α, NFR-1 and TFAM, thereby suppressing oxidative stress and generating more ATP for energy supplementation, which may be the underlying mechanism of WOPs.

Hence, these finding indicate that WOPs might attenuate aging-related muscle loss, both in terms of muscle mass and physical performance in vivo. Moreover, it has been shown that mitochondrial function can be improved by upregulating the expression of AMPK, PGC-1α, NRF-1 and TFAM, as well as mitochondrial DNA content in the gastrocnemius tissue of mice. 

Our study had several limitations. Firstly, the dynamic intervention effect is still unclear, as the results only concern one time point in sampling. Secondly, there is no evidence regarding mitochondrial morphology since we did not carry out electron microscopy examination. Thirdly, although the results of this study provided good insight in vivo, the direct results highlighting the efficiency of WOPs on sarcopenia dose extrapolation still has a long way to go. Therefore, clinical trials and primary cell cultures should be carried out further, which will be helpful for confirming the observed effects, interpreting the mechanism on a deeper level, and elucidating human applications.

## 5. Conclusions

The present findings indicate that effective WOPs effectively improve muscle loss, as well as the physical function in SAMP8 mice. Moreover, WOP intervention can decrease the levels of the inflammatory factors and increase expressions of AMPK, PGC-1α, NRF-1, and TFAM, as well as mitochondrial DNA content. Therefore, we assume that the alleviative effect of WOPs may be related to improving mitochondria function via AMPK/PGC-1α/NRF-1/TFAM, and reducing the expression of inflammatory factors, in gastrocnemius tissue. Thus, the findings of this study suggest that WOP supplementation is a potential strategy for use in sarcopenia prevention and treatment. 

## Figures and Tables

**Table 1:** Body composition and gastrocnemius tissue mass in mice.

	Lean Mass (%Body Weight)	Gastrocnemius (%Body Weight)
SAMR1	85.41 ± 0.41	1.37 ± 0.13
Control	81.22 ± 0.23 *	0.98 ± 0.11 *
WOPs-LG	83.12 ± 0.30 ^#^	1.21 ± 0.14 ^#^
WOPs-MG	82.41 ± 0.12 *	1.26 ± 0.12 ^#^
WOPs-HG	84.17 ± 0.19 ^#^	1.19 ± 0.09 ^#^

Data is expressed as mean ± SEM (*n* = 15 per group). *p* values were assessed using ANOVA-test. * *p* < 0.05 vs. SAMR1 and ^#^ *p* < 0.05 vs. Control. SAMR1, SAMR1 model control group; Control, SAMP8 age control group; WOPs(walnut oligopeptides )-LG, SAMP8-WOPs-(LG) at a dose of 110 mg/kg·bw; WOPs-MG, SAMP8-WOPs-MGat a dose of 220 mg/kg·bw; WOPs -HG, SAMP8-WOPs-HG at a dose of 440 mg/kg·bw.

## Data Availability

The data presented in this study are available on request from the corresponding author. The data are not publicly available due to privacy. The studies not involving humans.
